# Supplementary material for: Mutation of Basic Residues R283, R286, and K288 in the Matrix Protein of Newcastle Disease Virus Attenuates Viral Replication and Pathogenicity
Source: Int J Mol Sci. 2023 Jan 4;24(2):980. doi: 10.3390/ijms24020980 (PMC9864103; doi:10.3390/ijms24020980)
Supplement: Supplementary file 1 [file ijms-24-00980-s001.zip › ijms-2006583-supplementary.pdf]

Supplementary Materials

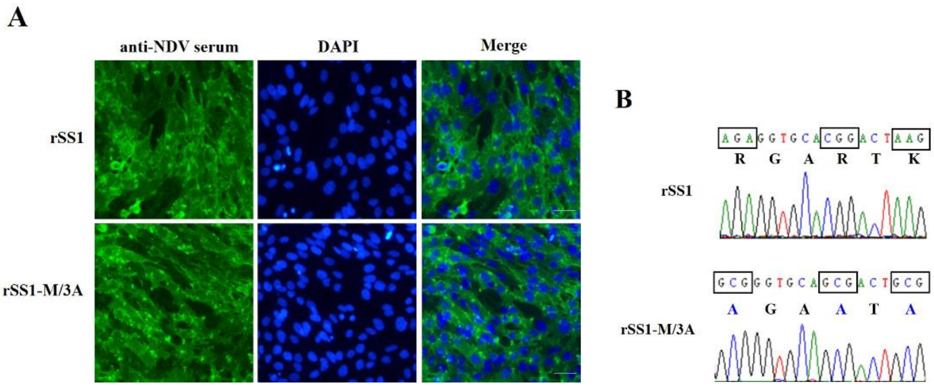

Figure S1. Identification of the rescued virus rSS1-M/3A by IFA and DNA sequencing. (A) Characterization of rSS1-M/3A in virus-infected DF-1 cells by IFA. (B) Nucleotide sequence analysis of the M/3A mutations introduced in the rSS1-M/3A *M* gene after serially passaged five times in 10-day-old SPF chicken eggs.

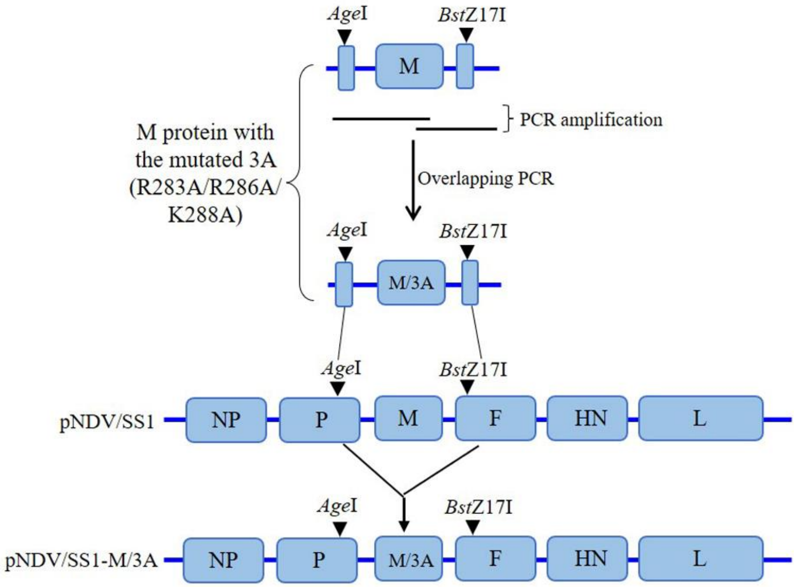

Figure S2. Schematic diagram of the mutation strategy for the construction of the full-length cDNA clone pNDV/SS1-M/3A.

Table S1. The information of primers used for the construction of recombinant plasmids.

Plasmid	Sense Primer (5'→3')	Anti-Sense Primer (5'→3')	Restriction Sites [a]
pCMV-HA-M(264-292)	*AATTC*GGCTCAATCTATCTGTTGGGCTCAGTGATGTGCTCGGGCCCTCTGTGCTTGTGAAGGCGAGAGGTGCACGGACTAAGCTACTTGCTCCTTC	*TCGAG*AAGGAGCAAGTAGCTTAGTCCGTGCACCTCTCGCCTTCACAAGCACAGAGGGCCCGAGCACATCACTGAGCCCAACAGATAGATTGAGCCG	*Eco*R I /*Xhol* I
pCMV-HA-M(289-313)	*AATTC*GGCTACTTGCTCCTTTCTTCTCTAGCAGTGGGACAGCCTGCTATCCTATAGCAAATGCCTCTCCCCAGGTTGCCAAGTC	*TCGAG*ACTTGGCAACCTGGGGAGAGGCATTTGCTATAGGATAGCAGGCTGTCCCACTGCTAGAGAAGAAAGGAGCAAGTAGCCG	*Eco*R I /*Xhol* I
pCMV-HA-M(264-280)	*AATTC*GGCTCAATCTATCTGTTGGGCTCAGTGATGTGCTCGGGCCCTCTGTGCTTGTGTC	*TCGAG*ACACAAGCACAGAGGGCCCGAGCACATCACTGAGCCCAACAGATAGATTGAGCCG	*Eco*R I /*Xhol* I
pCMV-HA-M(281-288)	*AATTC*GGAAGGCGAGAGGTGCACGGACTAAGTC	*TCGAG*ACTTAGTCCGTGCACCTCTCGCCTTCCG	*Eco*R I /*Xhol* I
pCMV-HA-M/mt1	*AATTC*GGCTCAATCTATCTGTTGGGCTCAGTGATGTGCTCGGGCCCTCTGTGCTTGTGGCGGCGAGAGGTGCACGGACTAAGCTACTTGCTCCTTTCTTCTCTAGCAGTGGGACAGCCTGCTATCCTATAGCAAATGCCTCTCCCCAGGTTGCCAAGTC	*TCGAG*ACTTGGCAACCTGGGGAGAGGCATTTGCTATAGGATAGCAGGCTGTCCCACTGCTAGAGAAGAAAGGAGCAAGTAGCTTAGTCCGTGCACCTCTCGCCGCCACAAGCACAGAGGGCCCGAGCACATCACTGAGCCCAACAGATAGATTGAGCCG	*Eco*R I /*Xhol* I
pCMV-HA-M/mt2	*AATTC*GGCTCAATCTATCTGTTGGGCTCAGTGATGTGCTCGGGCCCTCTGTGCTTGTGAAGGCGGCGGGTGCACGGACTAAGCTACTTGCTCCTTTCTTCTCTAGCAGTGGGACAGCCTGCTATCCTATAGCAAATGCCTCTCCCCAGGTTGCCAAGTC	*TCGAG*ACTTGGCAACCTGGGGAGAGGCATTTGCTATAGGATAGCAGGCTGTCCCACTGCTAGAGAAGAAAGGAGCAAGTAGCTTAGTCCGTGCACCCGCCGCCTTCACAAGCACAGAGGGCCCGAGCACATCACTGAGCCCAACAGATAGATTGAGCCG	*Eco*R I /*Xhol* I
pCMV-HA-M/mt3	*AATTC*GGCTCAATCTATCTGTTGGGCTCAGTGATGTGCTCGGGCCCTCTGTGCTTGTGAAGGCGAGAGCGGCACGGACTAAGCTACTTGCTCCTTTCTTCTCTAGCAGTGGGACAGCCTGCTATCCTATAGCAAATGCCTCTCCCCAGGTTGCCAAGTC	*TCGAG*ACTTGGCAACCTGGGGAGAGGCATTTGCTATAGGATAGCAGGCTGTCCCACTGCTAGAGAAGAAAGGAGCAAGTAGCTTAGTCCGTGCCGCTCTCGCCTTCACAAGCACAGAGGGCCCGAGCACATCACTGAGCCCAACAGATAGATTGAGCCG	*Eco*R I /*Xhol* I
pCMV-HA-M/mt4	*AATTC*GGCTCAATCTATCTGTTGGGCTCAGTGATGTGCTCGGGCCCTCTGTGCTTGTGAAGGCGAGAGGTGCAGCGACTAAGCTACTTGCTCCTTTCTTCTCTAGCAGTGGGACAGCCTGCTATCCTATAGCAAATGCCTCTCCCCAGGTTGCCAAGTC	*TCGAG*ACTTGGCAACCTGGGGAGAGGCATTTGCTATAGGATAGCAGGCTGTCCCACTGCTAGAGAAGAAAGGAGCAAGTAGCTTAGTCGCTGCACCTCTCGCCTTCACAAGCACAGAGGGCCCGAGCACATCACTGAGCCCAACAGATAGATTGAGCCG	*Eco*R I /*Xhol* I
pCMV-HA-M/mt5	*AATTC*GGCTCAATCTATCTGTTGGGCTCAGTGATGTGCTCGGGCCCTCTGTGCTTGTGAAGGCGAGAGGTGCACGGGCGAAGCTACTTGCTCCTTTCTTCTCTAGCAGTGGGACAGCCTGCTATCCTATAGCAAATGCCTCTCCCCAGGTTGCCAAGTC	*TCGAG*ACTTGGCAACCTGGGGAGAGGCATTTGCTATAGGATAGCAGGCTGTCCCACTGCTAGAGAAGAAAGGAGCAAGTAGCTTCGCCCGTGCACCTCTCGCCTTCACAAGCACAGAGGGCCCGAGCACATCACTGAGCCCAACAGATAGATTGAGCCG	*Eco*R I /*Xhol* I

pCMV-HA-M/mt6	*AATTC*GGCTCAATCTATCTGTTGG<br>GCTCAGTGATGTGCTCGGGCCCTC<br>TGTGCTTGTGAAGGCGAGAGGTG<br>CACGGACTGCGCTACTTGCTCCTT<br>TCTTCTCTAGCAGTGGGACAGCCT<br>GCTATCCTATAGCAAATGCCTCTC<br>CCCAGGTTGCCAAGTC	*TCGAG*ACTTGGCAACCTGGGGAGAG<br>GCATTTGCTATAGGATAGCAGGCTG<br>TCCCACTGCTAGAGAAGAAAGGAG<br>CAAGTAGCGCAGTCCGTGCACCTCT<br>CGCCTTCACAAGCACAGAGGGCCC<br>GAGCACATCACTGAGCCCAACAGA<br>TAGATTGAGCCG	*Eco*R I /*XhoI* I
pCMV-HA-M/mt7	*AATTC*GGCTCAATCTATCTGTTGG<br>GCTCAGTGATGTGCTCGGGCCCTC<br>TGTGCTTGTGAAGGCGGCGGGTG<br>CAGCGACTGCGCTACTTGCTCCTT<br>TCTTCTCTAGCAGTGGGACAGCCT<br>GCTATCCTATAGCAAATGCCTCTC<br>CCCAGGTTGCCAAGTC	*TCGAG*ACTTGGCAACCTGGGGAGAG<br>GCATTTGCTATAGGATAGCAGGCTG<br>TCCCACTGCTAGAGAAGAAAGGAG<br>CAAGTAGCGCAGTCGCTGCACCCGC<br>CGCCTTCACAAGCACAGAGGGCCC<br>GAGCACATCACTGAGCCCAACAGA<br>TAGATTGAGCCG	*Eco*R I /*XhoI* I
pCMV-HA-M/3A	TT*GAATTC*GGATGGACTCATCCAG<br>GAC	CGCAGTCGCTGCACCCGCCGCCTTC<br>ACAAGCACAGAGGGCCC	*Eco*R I
	AAGGCGGCGGGTGCAGCGACTGC<br>GCTACTTGCTCCTTTCTTCTCTAGC	GCA*CTCGAG*TTATTTCCTGAAAGG	*XhoI* I
pNDV/SS1-M/3A	ACA*ACCGGT*GCAGCACCCTTCTGA<br>ATTG	CGCAGTCGCTGCACCCGCCGCCTTC<br>ACAAGCACAGAGGGCCC	*Age* I
	AAGGCGGCGGGTGCAGCGACTGC<br>GCTACTTGCTCCTTTCTTCTCTAGC	GTG*GTATAC*ATTGACTGCCTTATCTC<br>CTG	*Bst*Z17 I

[a]: Restriction sites are given in italics and underlines.
